# Crosstalks between inflammasome and autophagy in cancer

**DOI:** 10.1186/s13045-020-00936-9

**Published:** 2020-07-23

**Authors:** Chaeuk Chung, Wonhyoung Seo, Prashanta Silwal, Eun-Kyeong Jo

**Affiliations:** 1 Division of Pulmonary and Critical Care, Department of Internal Medicine, Chungnam National University School of Medicine, Daejeon, 35015 Korea; 2 Infection Control Convergence Research Center, Chungnam National University School of Medicine, Daejeon, 35015 Korea; 3 Department of Microbiology, Chungnam National University School of Medicine, Daejeon, 35015 Korea; 4 Department of Medical Science, Chungnam National University School of Medicine, Daejeon, 35015 Korea

**Keywords:** Inflammasome, Autophagy, Mitophagy, Mitochondrial ROS, Cancer

## Abstract

Both inflammasomes and autophagy have important roles in the intracellular homeostasis, inflammation, and pathology; the dysregulation of these processes is often associated with the pathogenesis of numerous cancers. In addition, they can crosstalk with each other in multifaceted ways to influence various physiological and pathological responses, including cancer. Multiple molecular mechanisms connect the autophagy pathway to inflammasome activation and, through this, may influence the outcome of pro-tumor or anti-tumor responses depending on the cancer types, microenvironment, and the disease stage. In this review, we highlight the rapidly growing literature on the various mechanisms by which autophagy interacts with the inflammasome pathway, to encourage additional applications in the context of tumors. In addition, we provide insight into the mechanisms by which pathogen modulates the autophagy-inflammasome pathway to favor the infection-induced carcinogenesis. We also explore the challenges and opportunities of using multiple small molecules/agents to target the autophagy/inflammasome axis and their effects upon cancer treatment. Finally, we discuss the emerging clinical efforts assessing the potential usefulness of targeting approaches for either autophagy or inflammasome as anti-cancer strategies, although it remains underexplored in terms of their crosstalks.

## Background

Autophagy is an intracellular catabolic process and plays a crucial role in the maintenance of homeostasis in a variety of biological processes. Depending on the disease stage and the tumor microenvironment, autophagy plays a dual role in cancer; it can either promote oncogenesis or suppress tumor growth. In most cancers, autophagy facilitates tumorigenesis by regulating mitochondrial quality control and the supply of nutrients required for cancer cell growth under nutrient-deprived conditions [1–4]. However, the induction of autophagy may suppress tumor growth through maintaining cellular integrity, preventing cellular damage, and attenuating cell stemness [4, 5]. Inflammasomes are large protein complexes required for the secretion of mature interleukin (IL)-1β and IL-18, and pyroptosis, an inflammatory, caspase-1-dependent form of programmed cell death [6]. The transcriptional regulation of inflammasome-associated pattern recognition receptors plays a critical role in cancer [7]. Dysregulation of NOD-like receptor (NLR) signaling is strongly linked to chronic inflammation and subsequent tumor development [8, 9]. However, inflammasome activation is also associated with a lower risk of colitis-associated colon cancer [10].

Apart from its role in diverse physiological and pathological conditions, the crosstalk between autophagy and inflammasomes is crucial in the pathogenesis of cancer [6, 11]. In general, autophagy is regarded as a safety mechanism counteracting hyperactivation of inflammasomes and chronic inflammation-induced cancer [12]. Defects in canonical autophagy or mitophagy can lead to pathological responses and necrosis, promoting chronic inflammation and tumorigenesis [12, 13]. Additionally, autophagy levels have been associated with cell death fate and cell clearance [12]. Autophagic cancer cell death can trigger autocrine or paracrine ATP signaling through purinergic receptors, activating NOD-, LRR-, and pyrin domain-containing protein 3 (NLRP3) inflammasome and IL-1β secretion [14]. In particular, ATP derived from dying cells is a strong mediator of pro-inflammatory responses in macrophages found in the tumor microenvironment, augmenting anti-tumor immune responses [14]. Thus, autophagy regulation is critical for tumor immune surveillance and cancer cell death [12].

In this review, we summarize the current knowledge regarding the interplay between autophagy and inflammasome activation in cancer. Particularly, we provide a brief overview of autophagy, mitophagy, and inflammasome pathways and outline various signaling molecules and molecular pathways that regulate the crosstalk between two processes to get more insights into their implications in cancers. In addition, we discuss several findings that pathogens modulate autophagy-inflammasome axis to facilitate infection-induced carcinogenesis. We also summarize the promising anti-cancer pharmacological agents that have been tested in vitro or in vivo and provide examples of clinical translation.

### Overview of autophagy and inflammasomes

Autophagy plays a dynamic role in different stages of tumorigenesis, either promoting or suppressing tumor development and progression [15, 16]. The molecular mechanisms underlying autophagy and selective autophagy have been comprehensively reviewed previously [17–19]. Herein, we provide a brief overview of autophagy and mitophagy, and inflammasomes, before dissecting the interactions between two processes in the context of cancer.

### Autophagy

**Fig. 1:**
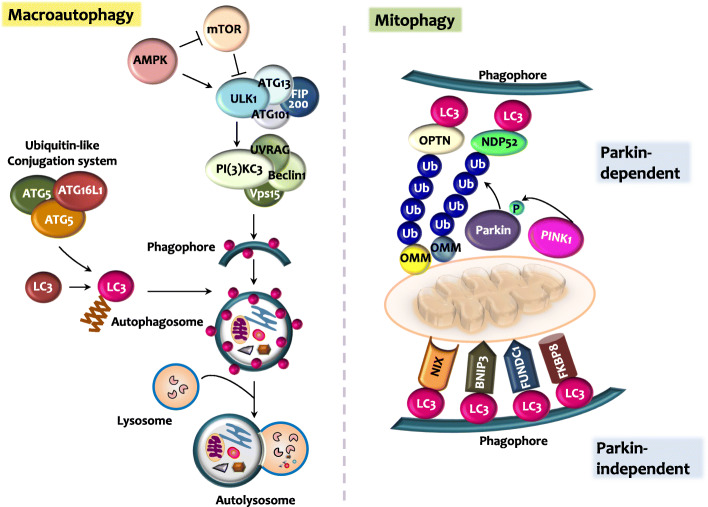
Overview of macroautophagy and mitophagy. Autophagy has dual roles in cancer, depending on the disease stage and tumor microenvironment. A summary of macroautophagy (autophagy) and mitophagy is shown. Autophagy can be divided into three steps: initiation, elongation, and maturation. In each step, several key players participate in the formation of phagophore (initiation), autophagosome elongation, and maturation (fusion of autophagosomes and lysosomes). In mitophagy, dysfunctional mitochondria are recognized by Parkin-dependent or Parkin-independent pathways. Ubiquitin, ubiquitin-binding proteins, and autophagy receptors, such as p62, NBR1, NDP52, and OPTN, are involved in the Parkin/PINK1-dependent mitophagy activation. In the Parkin-independent mitophagy pathway, several mitophagy receptors (Nix/BNIP3L, BNIP3, FUNDC1, BCL2L13, and FKBP8) direct damaged mitochondria to the LC3-mediated autophagy machinery

Autophagy is a “self-eating” process involving the degradation of, or dysfunctional cellular components through, fusion with lysosomes [20]. Several types of autophagy have been described, including macroautophagy, microautophagy, and chaperone-mediated autophagy (CMA) [21]. Macroautophagy is regarded as the canonical autophagy and involves a network of autophagy proteins that mediate the non-selective bulk degradation process (Fig. 1) [21, 22]. During this process, nutrient deprivation and metabolic stress trigger the activation of 5′-AMP-activated protein kinase (AMPK), which in turn activates unc-51-like autophagy-activating kinase (ULK). The ULK1 complex, consisting of ULK1, FIP200, ATG13, and ATG101, activates Beclin-1. Beclin-1 activation induces the formation and activation of class III phosphoinositide 3-kinase (PI3K) VPS34 complex, which is composed of Beclin-1, VPS34, VPS15, and ATG14L [23]. The VPS34 complex generates PI3P (phosphatidylinositol 3-phosphate)-rich subdomains on the endoplasmatic reticulum (ER) or ER-mitochondria contact sites, where PI3P-binding proteins can recruit the E3-like complex ATG16L1 (ATG5-12-16 L1). Subsequently, the ATG16L1 complex promotes the conjugation of ubiquitin-like molecule LC3 to phosphatidylethanolamine (PE) to generate LC3-PE (LC3-II), which is essential for the formation of double-membrane autophagosomes through membrane tethering and fusion. Autophagosomes are then fused with lysosomes, mediated by tethering factors, SNAP receptors (SNAREs), and phospholipids [24]. Finally, the cargo is degraded and recycled in the lysosomes (Fig. 1) [21].

### Mitophagy

Selective autophagy involves the targeted degradation of specific cellular components and organelles. It has become evident that the ubiquitin, ubiquitin-binding proteins, and autophagy receptors, including sequestosome-1/p62, neighbor of BRCA1 (NBR1), nuclear dot protein 52 (NDP52), and optineurin (OPTN) are vital for the activation of selective autophagy in a context-dependent manner [17].

Mitophagy is a type of selective autophagy, playing an essential role in the maintenance of mitochondrial homeostasis [25]. Mitochondrial perturbation, reactive oxygen species (ROS), and oxidative stress can trigger mitophagy to degrade dysfunctional mitochondria as a mitochondrial quality control mechanism [25]. The PTEN-induced putative kinase protein 1 (PINK1) activates the E3 ubiquitin ligase Parkin, which translocates from the cytosol into the damaged mitochondria to ubiquitinate outer mitochondrial membrane (OMM) proteins, such as mitofusin (MFN) and voltage-dependent protein channel 1 (VDAC1). Furthermore, several mitophagy receptors have been identified, including BCL2/adenovirus E1B 19 kDa protein-interacting protein 3-like (Nix/BNIP3L) [26], BCL2/adenovirus E1B 19 kDa protein-interacting protein 3 (BNIP3) [27], FUN14 domain-containing 1 (FUNDC1) [28], BCL2-like 13 (BCL2L13) [29], and FK506-binding protein 8 (FKBP8) [30]. Upon stress-induced activation, these mitophagy receptors are anchored in the OMM, recruiting ATG8 family members to the damaged mitochondria (Fig. 1) [31]. The expression of several mitophagy receptors and mediators is dysregulated in cancer, pinpointing the critical anti-tumor roles of mitophagy [32].

### Overview of inflammasome activation

**Fig. 2:**
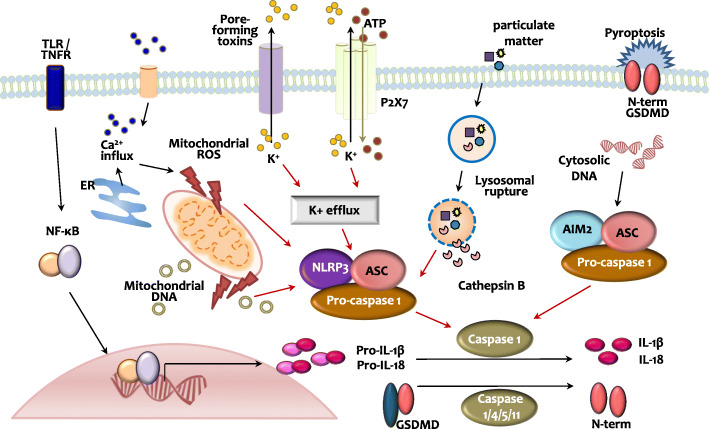
NLRP3 and AIM2 inflammasome pathways. The NLRP3 inflammasome activation is mediated through two signals: Toll-like receptor (TLR)/tumor necrosis factor receptor (TNFR)-mediated NF-κB pathway activation or inflammasome complex assembly (NLRP3, ASC, and pro-caspase-1) triggered by particulate matter (lysosomal destabilization or cathepsin B release), mitochondrial ROS generation, intracellular calcium influx, or potassium efflux. The activated NLRP3 inflammasome promotes IL-1β and IL-18 maturation and induces pyroptotic cell death (osmotic lysis of cells). AIM2 inflammasome assembly is induced by the recognition of cytosolic DNA and leads to pyroptosis and IL-1β/IL-18 maturation

Inflammasomes are multiprotein complexes; upon formation and activation, they activate caspase-1, which promotes pyroptotic cell death (pyroptosis), as well as the maturation of IL-1β and IL-18 [33]. The innate sensor molecules of inflammasomes include different NLRs, absent in melanoma 2 (AIM2) and Pyrin. NLRP3 is the most well-characterized inflammasome, and the molecular and cellular events leading to its activation have been extensively reviewed elsewhere [34]. Herein, we briefly outline the mechanisms underlying the activation of NLRP3 and AIM2 inflammasomes (Fig. 2), as well as their crosstalk with autophagy in the context of cancer.

NLRP3 inflammasomes are sensor protein complexes composed of NLRP3 and the adaptor protein apoptosis-associated speck-like protein containing a CARD (ASC). ASC recruits pro-caspase-1, which is subsequently cleaved to caspase-1. The latter catalyzes the proteolytic maturation of IL-1β and IL-18 [34]. Although the mechanisms underlying NLRP3 inflammasome activation are not fully understood, various pathogens and danger signals have been shown to trigger its activation [33, 34]. NLRP3 inflammasome activation is believed to occur in two steps: the first signal (priming) induces NLRP3 activation and pro-IL-1β expression through nuclear factor kappa-light-chain-enhancer of activated B cells (NF-κB) signaling and the second signal (activation) promotes NLRP3 inflammasome assembly. Due to the structural diversity of its ligands, NLRP3 inflammasome activation does not seem to be structure-dependent [34]. Instead, it is likely to be mediated via several molecular signaling pathways, including K^+^ efflux, Ca^2+^ signaling, mitochondrial ROS generation, and lysosomal rupture (Fig. 2) [33]. AIM2 is a member of the hematopoietic interferon-inducible nuclear proteins with a 200-amino-acid repeat (HIN-200) family; it induces inflammasome formation through recognition of aberrant and cytosolic double-stranded DNA (dsDNA). AIM2 inflammasomes also contain ASC and caspase-1, which are responsible for the maturation of IL-1β and IL-18 (Fig. 2) [35].

The activation of both NLRP3 and AIM2 inflammasomes results in inflammatory cell death, also known as pyroptosis, through the cleavage of gasdermin D (GSDMD) and activation of IL-1β and IL-18 (Fig. 2) [36, 37]. Pyroptosis can be induced by caspase-1/4/5/11, and caspase-4/5/11-mediated pyroptosis activates the noncanonical inflammasome pathway [36, 37]. Caspase-1-mediated pyroptosis is triggered by NLRP3 or AIM2 inflammasomes and involves the release of the pore-forming N-terminal fragment of GSDMD (GSDMD-NT) in the plasma membrane; the formation of pores facilitates the secretion of inflammatory cytokines and osmotic lysis of the cells (Fig. 2). These responses, and the release of IL-1β and IL-18, in particular, result in cell death and tissue damage [36, 37]. Recent studies highlighted that the modulation of GSDMD and pyroptosis can influence all stages of carcinogenesis, i.e., tumor cell proliferation, invasion, and metastasis [37, 38]. Both gasdermin E (GSDME) and GSDMD, important pyroptosis substrates, are also emerging targets as prognostic biomarkers for the management of various cancers [37, 39]. Small molecules that trigger or inhibit pyroptosis can lead to inhibition of tumor cells [38, 39]. However, it has been largely uncharacterized whether and how both GSDMD and GSDME are associated with the autophagy process while regulating progression or inhibition of tumorigenesis.

### Crosstalk between inflammasome activation and autophagy in cancer

**Fig. 3:**
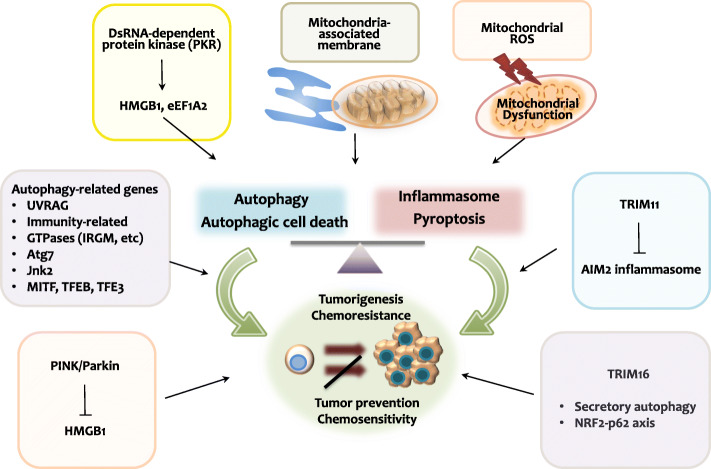
Crosstalk between autophagy and inflammasome activation in cancer. The crosstalk between autophagy and inflammasome activation regulates multiple physiological and pathological responses, including cancer. Mitochondrial dysfunction and mitochondrial ROS generation can activate autophagy/mitophagy, as well as act as the second signal for inflammasome activation and pyroptosis. Dysfunctional autophagy results in excessive mitochondrial oxidative stress, leading to autophagic cell death, inflammasome activation, and pyroptosis. Elevated mitochondrial ROS levels can also promote oncogenesis, chemoresistance, and metastasis. Furthermore, mitochondria-associated membranes at the ER-mitochondria contact sites are signaling hubs for mitochondrial Ca^2+^ transfer from the ER to mitochondria through IP3R3s, mediating NLRP3 inflammasome activation in response to mitochondrial damage. IP3R3s are upregulated in various cancers

Accumulating evidence supports the importance of crosstalk between inflammasome activation and autophagy in numerous biological and pathological processes, particularly in infection and inflammation. Recent studies suggest that defects in this interplay have been linked to cancers, i.e., tumorigenesis, cancer stemness, and resistance to anti-cancer therapies. In the following sections, we describe several mechanisms and key players participating in the interplay between autophagy and inflammasomes to provide insight into the tumorigenesis, metastasis, and treatment of cancers (Fig. 3). Increasing understanding of the molecular mechanisms for the autophagy-inflammasome axis could aid in the design of improved anti-cancer therapeutics from the bench works to the clinical settings.

### Mitochondrial dysfunction and ROS

Mitochondrial ROS are key upstream regulators of NLRP3 inflammasomes [40] and autophagy [41, 42]. Balance in the activation of inflammasomes and autophagy is essential for mitochondrial homeostasis (Fig. 3) [41, 42]. Dysfunctional autophagy results in mitochondrial oxidative stress and damage, leading to autophagic cell death, exaggerated activation of inflammasome, and pyroptosis [43, 44].

In cancer cells, intracellular redox signals modulate tumor progression and chemoresistance [45]. Imbalance in mitochondrial Ca^2+^ or ROS levels is frequently observed in most human cancers [45, 46]. Aberrant production of mitochondrial ROS can promote cancer cell proliferation, migration, or survival/apoptosis, depending on the context [47, 48]. Enhanced metabolism and increased ATP production via the electron transport chain are imperative for tumor progression and metastasis, with increasing mitochondrial ROS levels at the same time [47, 48]. Furthermore, since ATP-driven multidrug efflux is essential for the development of chemoresistance in cancer cells, hyperactivation of the electron transport chain and increased ROS production contribute to therapy resistance [45]. In addition to the enhanced mitochondrial ROS generation, ROS detoxification pathways are highly activated in tumors [45].

**Fig. 4:**
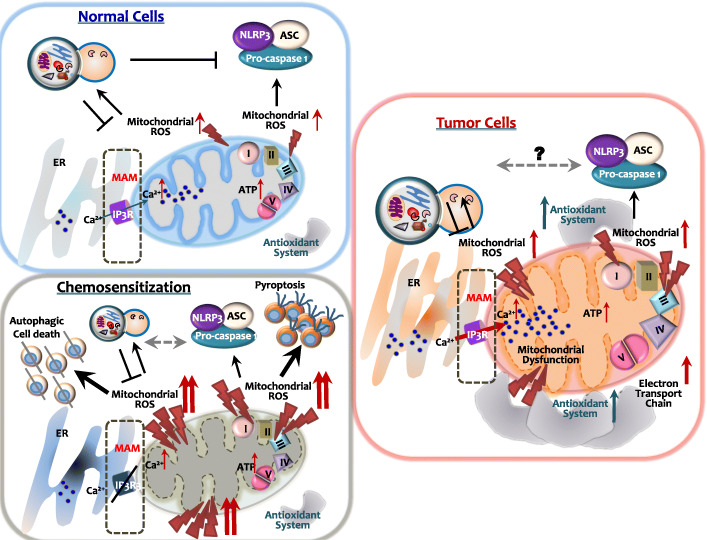
The role of mitochondrial ROS in the regulation of the autophagy/inflammasome axis at MAMs in non-malignant cells, cancer cells, and chemosensitized cells. MAMs are signaling hubs, playing crucial roles in the crosstalk between autophagy and inflammasome activation, as well as intracellular Ca^2+^ signaling, mitochondrial lipid metabolism, and bioenergetics. Mitochondrial dysfunction and subsequent mitochondrial ROS generation activate autophagy/mitophagy, which negatively regulates NLRP3 inflammasome activation in non-malignant cells. Cancer cells are characterized by elevated mitochondrial ROS levels, accompanied by the upregulation of antioxidant machinery components. The role of mitochondrial ROS in the crosstalk between autophagy and inflammasome activation in cancer cells remains unclear. In chemosensitized cells, excessive production of mitochondrial ROS results in autophagic and pyroptotic cell death. Although the role of IP3R in the regulation of the autophagy/inflammasome axis remains unknown, IP3R inhibition can suppress tumor growth

Moreover, excessive mitochondrial ROS generation during cancer treatment can induce a synergistic antitumor response during chemotherapy. A recent study showed that lung cancer apoptosis was increased in the condition of excessive mitochondrial fission and marked upregulation of mitochondrial ROS [49]. In addition, tumor necrosis factor-related apoptosis-inducing ligand (TRAIL) combination with gold nanoparticles led to a hyperactivation of mitochondrial fragmentation and mitochondrial dysfunction in non-small-cell lung cancer cells, thereby promoting apoptosis of cancer cells to TRAIL [50]. In another study, the anticancer responses of oxaliplatin were enhanced by combination with piperlongumine, a molecule promoting ROS in colorectal cancer [51]. In cholangiocarcinoma, a very aggressive cancer, mitochondrial division inhibitor-1 (Mdivi-1) sensitized cancer cells to cisplatin cytotoxicity with increased oxidative stress and inhibition of autophagosomes [52]. Emerging evidence suggests the effects of chemosensitizers in mitochondrial dysfunction and ROS generation to overcome chemoresistance in various cancer settings. Nevertheless, it is currently unclear whether hyper-activation of mitochondrial ROS by a variety of sensitizers directly or indirectly regulates the interplay between autophagy and inflammasomes in anti-cancer treatment. An ongoing paradigm of mitochondrial ROS generation in normal and cancer cells, as well as chemotherapeutic resistance and sensitization, is summarized in Fig. 4. A better understanding of the molecular mechanisms by which mitochondrial ROS link between autophagy and inflammasome activation in different types and stages of cancer cells may enable to offer improved treatment against chemoresistant tumors.

### Mitochondria-associated membranes

Mitochondria-associated membranes (MAMs) at the ER-mitochondria contact sites are crucial for the activation of NLRP3 inflammasomes and autophagy [15, 53, 54]. Importantly, autophagy activation at MAMs mediates the removal of dysfunctional or aged mitochondria, thereby inhibiting NLRP3 inflammasome activation [55]. The signaling events occurring at the ER-mitochondria contact sites appear to be important in determining the fate of the cell, regulating tumor progression [15].

MAMs are signaling hubs for intracellular Ca^2+^-dependent pathways that regulate lipid synthesis and mitochondrial bioenergetics [54]. Ca^2+^ transfer from the ER into mitochondria is considered critical for NLRP3 inflammasome activation by inducing mitochondrial damage [56]. In particular, type 3 inositol 1,4,5-trisphosphate (IP3) receptors (IP3R3s) located at MAMs mediate pro-apoptotic and anti-cancer effects [57]. Paradoxically, IP3R3 levels are elevated in cancer, and several IP3R3s have been implicated in oncogenesis and cancer cell survival [58–60]. Since many cancer cells rely on ER-mitochondrial Ca^2+^ fueling, inhibition of IP3Rs can suppress cancer cell proliferation and migration and promote cell death [61]. Although the mechanisms remain unclear, enhanced autophagy has been shown to mediate the anti-tumor effects of IP3R-targeting agents [62]. However, the role of IP3R modulation on inflammasome activation is yet to be elucidated. The role of mitochondrial ROS at MAMs for the regulation of the autophagy/inflammasome axis is illustrated in Fig. 4, in both non-malignant cells and cancer cells. Future studies are required to elucidate the relevance of IP3Rs in regulating autophagy, inflammasome activation, and cell death.

### Double-stranded RNA-dependent protein kinase

The translation of mRNAs is closely linked to cancer cell proliferation, as well as tumor progression and metastasis [63]. Phosphorylation of the translation initiation factor eIF2 at serine 51 (hereafter referred to as eIF2α-P) is imperative for mRNA translation and is primarily mediated by the double-stranded RNA (dsRNA)-dependent protein kinase (PKR) [63]. PKR is a multifunctional protein, regulating autophagy and inflammasome activation, as well as promoting the extracellular release of high-mobility group box 1 (HMGB1) protein (Fig. 3) [64]. Although PKR was thought to be a tumor suppressor, several pro-tumorigenic functions of PKR has been demonstrated in various cancers, including colon, breast, and liver cancer [65]. However, the present understanding about the role of PKR in the connection between autophagy and inflammasome is still preliminary in terms of cancer. An example was reported in hepatocellular carcinoma. PKR is upregulated in hepatitis C virus (HCV)-related hepatocellular carcinoma [66] and promotes cancer cell growth by activating the MAPK pathway [65]. In addition to hepatocellular carcinoma cell growth, PKR induces autophagy and inflammasome activation in an HMGB1-dependent manner [65].

PKR interacts with the translation elongation factor eEF1A2, promoting cell survival and other malignant characteristics in preneoplastic precursor cells [67]. Thus, translation inhibitors targeting eIF4E have been investigated as anti-cancer agents in various hematologic malignancies [63]. The drug plitidepsin, which inhibits the interaction between eEF1A2 and PKR, has been shown to induce cancer cell death by activating the extrinsic apoptosis pathway [67]. In addition, PKR/eIF2α-P axis inhibition showed anti-tumor effects against HER2-positive breast cancer and gastric cancer [68]. Moreover, the eIF2α-phosphatase inhibitor SAL003 potentiated the anti-tumor effects of trastuzumab in HER2-positive tumor cells [68]. eIF2α-P levels have been proposed as a prognostic marker in HER2-positive breast cancer patients treated with trastuzumab [68]. Given the multifaceted role for PKR in different tumor entities [65, 67], further investigation into the involvement of autophagy-inflammasome pathway in the development of PKR-targeting anti-cancer therapeutic interventions is warranted.

### Autophagy-related molecules

Numerous studies have shown that the autophagy gene *UVRAG* functions as a tumor suppressor. Notably, the use of transgenic mice that inducibly express *UVRAG* with a frameshift mutation (iUVRAG^FS^) uncovered several of the anti-tumor roles of UVRAG [69]. Importantly, the transgenic mice exhibited intestinal inflammatory responses and increased susceptibility to colitis-associated cancer in an NLRP3 inflammasome-dependent manner. In addition, iUVRAG^FS^ mice were more prone to spontaneous tumorigenesis, which was associated with age-related autophagy suppression [69].

Immunity-related GTPases (IRGs), a family of interferon (IFN)-inducible GTPases, are required for innate immune responses against intracellular bacteria and protozoa [70]. IRGM/Irgm1 is involved in autophagy and has been implicated in Crohn’s disease. Recent studies have demonstrated that IRGM is a key negative regulator of NLRP3 inflammasome activation by interacting with NLRP3 and ASC and subsequently inhibiting inflammasome assembly [71]. In line with this, IRGM mediates the autophagic degradation of NLRP3 components [72]. However, there is still relatively less known regarding the function of IRGM in cancer. In AGBL2-overexpressing hepatocellular carcinoma cells, IRGM-mediated autophagy promoted cancer cell survival and proliferation [73]. Recent reports revealed that IRGM was upregulated in human glioma and functioned in glioma cell proliferation and autophagy [74]. Other studies showed the involvement of ATG7 and JNK2 in the connection with the autophagy-inflammasome pathway in the experimental model of cancers [75, 76]. In a rat insulinoma cell line, ATG7 induced autophagy in response to palmitic acid. It also induced cathepsin B (CTSB) expression, enhancing NLRP3-mediated IL-1β secretion and lipotoxicity [75], suggesting a link between autophagy overactivation and susceptibility to diabetic inflammation. Additionally, JNK2 is involved in stress-induced mitophagy and prevents the hyperactivation of inflammasomes by targeting the tumor suppressor ARF [76].

Indeed, the activation of autophagy/mitophagy, in particular, lysosomal function, is potentially increasing cancer growth and aggressiveness, as it can regulate mitochondrial metabolism [1]. Several lysosomal activity-inhibitory agents are currently being tested as therapeutic strategies for a variety of cancers. Most of these agents target master regulators of autophagy and lysosomal biogenesis, such as MiT/TFE transcription factors (MITF, TFEB, or TFE3) [77]. These proteins are able to shuttle between lysosomes and nucleus to regulate transcriptional responses in response to the change of nutrient and growth factor, thus affecting cancer biology [70]. Furthermore, tumor-derived autophagosomes can strongly activate innate immune responses and NLRP3 inflammasomes in the absence of LPS priming and are, therefore, being tested as therapeutic cancer vaccines [78]. The autophagosomes isolated from cancer cells are named as defective ribosomal products in blebs (Dribbles) can induce strong T cell responses and activate antiben-presenting cells, thus promoting adaptive immune responses against tumor cells and viruses [78]. Hence, these studies suggest that autophagy/mitophagy-associated molecules may be the major players in both tumorigenesis and anti-tumor immune responses (Fig. 3). Although there are numerous autophagy-related genes, it is still in its infancy to understand the in vitro and in vivo function of these genes in variety of cancers. Further investigations into the underlying mechanisms by which autophagy-related genes may impact inflammasome activation and pyroptosis are also needed to optimize therapeutic strategy.

### PINK1/Parkin and HMGB1

Mounting evidence suggests the essential role of mitophagy in the prevention of inflammatory diseases and cancer. Notably, Parkin translocation is impaired in chronic obstructive pulmonary disease (COPD), leading to the accumulation of dysfunctional mitochondria [13]. Thus, defective mitophagy and mitochondrial dysfunction are believed to be involved in the development of COPD-associated lung cancer [13].

PINK1-PRKN/PARK2-mediated mitophagy has also been reported to suppress pancreatic tumor growth through the autophagic degradation of mitochondrial iron importers, including SLC25A37 and SLC25A28 [79, 80]. Genetic ablation of *Pink1* or *Park2* in mice increased susceptibility to oncogenic *Kras*-driven pancreatic cancer development [79]. Mechanistically, mitochondrial iron accumulation and subsequent AIM2 inflammasome activation promoted pancreatic tumorigenesis [79]. Notably, AIM2-induced HMGB1 release enhanced the expression of the immune checkpoint CD274/ programmed death-ligand 1 (PD-L1), accelerating pancreatic tumorigenesis [79]. Thus, the defects in mitophagy may amplify the inflammasome-mediated tumorigenesis through HMGB1, a key factor linking inflammasome and tumorigenesis.

Indeed, HMGB1 is involved in the expansion of hepatic progenitor cells and hepatic tumor progression in autophagy-deficient livers. Mechanistically, HMGB1 is released from autophagy-deficient hepatocytes through nuclear factor erythroid 2 (NFE2)-related factor 2 (NRF2)-mediated inflammasome activation [81]. These data highlight the role of HMGB1 in tumor progression and immunopathology, and that its extracellular release is dependent on inflammasome activation, which could be amplified in autophagy-defective cells (Fig. 3). However, there might be a positive feedback loop in the activation of inflammasome-autophagy axis by HMGB1. Previous study suggests that HMGB1-DNA complexes induced autophagy by binding to the receptor for advanced glycation endproducts (RAGE) and that autophagy negatively regulated AIM2 inflammasome activation [82]. Future innovative approaches based on mitophagy activation to tweak HMGB1 release and AIM2 inflammasome activation may be considered to develop potential therapeutic candidates against refractory tumors including pancreatic cancer.

### TRIM11 and TRIM16 as key links between autophagy and inflammasomes

Secretory autophagy, rather than conventional autophagy involving lysosomes, is essential for IL-1β secretion. The secretory autophagy cargo IL-1β is recognized by tripartite motif-containing protein 16 (TRIM16), and its secretion is mediated by the interaction of SEC22B, syntaxin 3, and syntaxin 4 [83, 84]. TRIM16 plays a key role in the regulation of the p62-KEAP1-NRF2 system, particularly in the NRF2 stabilization and p62 expression in response to oxidative/proteotoxic stresses [85]. In addition, TRIM16 is essential for the NRF2/p62-mediated autophagy/aggrephagy activation, promoting protein homeostasis (proteostasis) and cancer cell survival during oxidative/proteotoxic stress (Fig. 3) [85, 86]. This action of TRIM16 is required for the protection of HeLa cell apoptosis and death from toxic misfolded proteins in vitro and in vivo [85]. Although these data suggest that TRIM16 is a prosurvival protein through the regulation of autophagy, NRF2-p62, and ubiquitin system [85], it has not been characterized if TRIM16 is involved in the regulation of inflammasome activation and pyroptosis while suppressing cancer cell cytotoxicity.

It is largely unknown that the roles of other TRIM members in anti-cancer or pro-cancer responses. Among TRIM members, TRIM11 was identified as an essential negative regulator of AIM2 inflammasome activation through the induction of selective autophagy (Fig. 3) [87], suggesting a regulator candidate of tumor cell survival and death. Since AIM2 inflammasome-associated DNA-sensing pathways are closely related to tumorigenesis [88], future studies are warranted to assess the role of TRIM11 in cancer.

### Potential anti-cancer therapeutics targeting the crosstalk between autophagy and inflammasomes

Emerging evidence from in vitro and in vivo studies suggest the potential clinical value of autophagy-inflammasome axis modulators as anti-cancer agents. In the following sections, we summarize potential anti-cancer treatment strategies using agents that modulate the crosstalk between autophagy and inflammasome activation.

### Effects of autophagy-modulating agents on inflammasomes

Silibinin, an anti-cancer agent used in breast cancer patients, has been shown to suppress cancer cell migration and invasion by interfering with ROS generation and suppressing NLRP3 inflammasome activation [89]. Numerous studies have also highlighted the autophagy-modulating effects of silibinin [90–92]; therefore, silibinin is considered a promising therapeutic approach for various cancers. Ergosterol peroxide, a molecule isolated from the fungus *Phoma sp.*, strongly induced ROS-dependent autophagy and caspase-dependent apoptosis in human lung adenocarcinoma cells. Additionally, ergosterol peroxide treatment inhibited tumor cell proliferation and migration by attenuating NLRP3 inflammasome activity [93]. Ergosterol peroxide also synergized with the anti-tumor drug sorafenib, exerting strong cytotoxic effects in human lung adenocarcinoma cells [93]. Although ergosterol peroxide inhibited apoptosis in lung adenocarcinoma, it remains unclear whether ergosterol peroxide-induced autophagy suppresses inflammasome activity directly [93].

Poly (amidoamine) (PAMAM) dendrimers are a novel class of nanomaterials. Interestingly, they induced autophagy hepatocellular carcinoma HepG2 cells, negatively affecting cell survival [94]. The hydrophobic polyphenol curcumin exerted anti-tumor effects and activated autophagy in several cancers both in vitro and in vivo [95, 96]. Given its multifaceted roles in the regulation of inflammasome activation [97, 98], it would be interesting to assess whether the anti-cancer effects of curcumin are mediated by activation of autophagy or by modulation of inflammasomes and pyroptosis. Coptisine, a natural compound extracted from *Coptis chinensis*, exerted potent anti-cancer effects through activation of autophagy or NLRP3 inflammasomes [99–101]. Taken together, various small molecules and nanomaterials modulating autophagy and inflammasome activation are currently being tested as anti-cancer agents for numerous cancer types.

Resveratrol, a natural polyphenolic phytoalexin, may play a protective role against cancer, particularly colorectal and skin cancer [102]. Interestingly, resveratrol activates and inhibits autophagy in colorectal cancer and skin cancer, respectively [103, 104]. Resveratrol can induce apoptosis and inhibit angiogenesis, thereby suppressing tumor growth and metastasis [105]. Importantly, resveratrol induced autophagy in various in vitro and in vivo disease models, by activating AMPK [106–109]. However, the mechanisms underlying the autophagy- and inflammasome-modulating effects of resveratrol in the context of cancer remain unclear. Small molecules, including GL-V9 (AMPK activator), exhibited strong anti-inflammatory effects by activating autophagy and inducing NLRP3 degradation, preventing colitis-associated colorectal cancer through [110]. The small molecule andrographolide prevented colitis progression and colon cancer development by suppressing the activation of NLRP3 inflammasomes. Andrographolide also inhibited the mammalian target of rapamycin (mTOR) pathway and induced mitophagy in macrophages, subsequently inhibiting NLRP3 inflammasomes [111]. Therefore, targeting the AMPK-mTOR pathway may serve as a promising therapeutic strategy for inflammation-associated cancers. Autophagy-modulating agents and their influences on inflammasomes and cancer are summarized in Table 1.

**Table 1:** Agents/small molecules of linking autophagy to inflammasomes in cancers

Agents	Cell/tissue	Mechanism	Outcome	Ref.
Silibinin	Breast cancer (MDA-MB-231)	Impairment of mitochondrial dynamics; Reduction of ROS generation and inhibition of NLRP3 inflammasome	Reduction of migration and invasion of tumor cell	[89]
	Brain cancer (A172, SR)	Inhibition of the mTOR pathway and upregulation of LC3 II expression	Increased apoptosis (amplified by autophagy inhibition)	[91]
	Salivary gland cancer (ACC-M)	Enhancement of LC3 expression	Inhibition of tumor cell proliferation and metastasis	[92]
Ergosterol peroxide	Non-small cell lung cancer (A549)	ROS-mediated autophagy and apoptosis; Inhibition of NLRP3 inflammasome; Downregulation of EGFR, Akt1, mTOR, and NF-κB	Increased apoptosis (amplified by autophagy inhibition)	[93]
Poly-amidoamine	Hepatocellular carcinoma (HepG2)	ROS-mediated autophagy and apoptosis; Activation of autophagy by inhibition of Akt/mTOR pathway; Upregulation of Inflammasome-related gene	Increased apoptosis (amplified by autophagy inhibition)	[94]
Coptisine	Hepatocellular carcinoma (HepG2, MHCC97-L)	Activation of autophagy through Beclin-1 and inhibition of mTOR signaling (by Berberine, structural homology of Coptisine)	Anti-cancer effect	[99]
	Bone marrow-derived macrophage, THP-1, Murine 3T3L-1	Inhibition of NLRP3 inflammasome via AMPK-dependent autophagy activation (by Berberine, structural homology of Coptisine)	Anti-inflammatory effect in adipose tissue macrophages	[101]
Curcumim	Melanoma (A375,C8161)	Inhibition of the Akt/mTOR/p70S6K pathway; autophagy activation	Anti-cancer effect	[96]
	Mesothelioma (LP9, HMESO, H2595, H2461)	Activation of NLRP3 inflammasome-mediated pyroptosis via ROS-dependent manner; Downregulation of NLRP3 inflammasome-related genes	Anti-cancer effect; Inhibition of inflammation	[98]
Resveratrol	Skin cancer (A431)	Aberration of autophagy and inhibition of autolysosome formation; Inhibition of mTORC2 by downregulation of Rictor expression	Preventive effect against tumorigenesis	[103]
	Colon cancer (HT-20,COLO201)	ROS-mediated activation of caspase-3, casepase-8, and elevation of LC3 II	Anti-cancer effect	[104]
	Human aortic endothelium	Reduction of intracellular ROS via autophagy through AMPK-mTOR	Protective autophagy	[106]
	Spinal cord	Activation of AMPK; inhibition of mTOR signaling pathway	Neuroprotective autophagy	[107]
	Spinal cord	Upregulation of SIRT1, p-AMPK, Beclin-1, LC3-B, and Bcl-2 expression	Neuroprotective autophagy	[108]
	Human peritoneal mesothelium	Activation of the AMPK pathway and inhibition of NLRP3 inflammasome in ROS stress condition of PMCs	Inhibition of peritoneal inflammation	[109]
GL-V9	Colon cancer, THP-1, bone marrow-derived macrophages	Activation of AMPK-ULK1 pathway; Degradation of NLRP3 inflammasome via autophagy	Protective effect against colitis; inhibition of colitis-induced cancer	[110]
Andrographo-lide	Colon cancer, THP-1, peritoneal macrophage, bone marrow-derived macrophage	Inhibition of PI3K/Akt1/mTOR/S6 kinase 1 pathway; Interruption of NLRP3 inflammasome assembly	Protective effect against colitis; inhibition of colitis-induced cancer	[111]

*SIRT1* Sirtuin 1, *Bcl2* B cell lymphoma 2, *PMC* peritoneal mesothelium cell

### Dual activators of inflammasomes and autophagy

Numerous agents have been reported to activate autophagy and inflammasomes, in addition to inhibiting tumorigenesis. The antimalarial drug dihydroartemisinin (DHA) activated AIM2/caspase-1 inflammasomes and induced autophagy, thereby suppressing hepatocellular carcinoma growth [112]. Additionally, DHA enhanced ROS generation by inducing DNA damage, contributing to the activation of autophagy [112]. Furthermore, the anti-tumor effects of mevalonate metabolism inhibitors, including statins and bisphosphonates, have been associated with their ability to suppress protein prenylation and thereby activate inflammasomes and autophagy [113–115]. Moreover, a recent study showed that the *Trillium tschonoskii* maxim saponin polyphyllin VI (PPVI) inhibited the proliferation of human non-small cell lung cancer cells by inducing pyroptosis, as well as apoptotic and autophagic cell death in a ROS-NLRP3 inflammasome-dependent way [116, 117]. However, the relationship between apoptotic and autophagic cell death after PPVI treatment remains unknown.

Recent studies showed that treatment with ceramide-1-phosphate (C1P) or ceramide-1-phosphate transfer protein inhibition (CPTP, involved in C1P trafficking) [118] induced autophagy and IL-1β/IL-18 secretion in human epithelial cells by activating NLRP3 inflammasomes [119]. Considering the role of C1P in the generation of arachidonic acid, a key mediator of cancer, it would be of high clinical relevance to determine the relationship between C1P-mediated eicosanoid production and C1P-mediated inflammasome and autophagy modulation.

Although the effects of the estrogen receptor (ER) ligand 17β-estradiol in autophagy have been controversial, several clinical trials are currently investigating its anti-cancer effects in breast cancer patients, in combination with autophagy modulators [120]. A recent study showed that 17β-estradiol suppressed hepatocellular carcinoma progression by inducing caspase-1-mediated pyroptosis and inhibiting autophagy [121]. However, estrogen/ERα-induced autophagy has been associated with papillary thyroid cancer cell survival [122]. Thus, further studies are required to elucidate the role of ER/17β-estradiol in tumorigenesis, as well as assess its potential as a therapeutic target in cancer. Small molecules activating both autophagy and inflammasomes are summarized in Table 2.

**Table 2:** Agents/small molecules for dual activation of autophagy and inflammasomes

Agents	Cell/tissue	Mechanism	Outcome	Ref.
Dihydroartemisinin (DHA)	Hepatocellular carcinoma (HepG2215)	Activation of ROS-mediated autophagy through inhibition of mTOR; Upregulation of AIM2 expression	Anti-cancer effects	[112]
Prenylation inhibitor	Prostatic cancer cell (PC3)	Activation of autophagy through inhibition of geranylgeranyl synthesis	Cell cycle arrest and inhibition of proliferation	[114]
	THP-1	Activation of NLRP3 inflammasome through ATP secretion and P2X7 activation via isoprenylation-dependent pathway	Not determined in cell survival/death	[115]
Polyphyllin VI	Non-small cell lung cancer (A549, H1299, PC-9)	Activation of ROS-induced NF-κB signaling and pyroptosis; NLRP3 inflammasome activation	Anti-cancer effect	[116]
	Non-small cell lung cancer (A549, H1299)	Activation of ROS-mediated autophagy through inhibition of mTOR; ATG7-dependent autophagic cell death	Anti-cancer effect (reduced by autophagy inhibition)	[117]
Ceramide-1-phosphate (C1P) transfer protein	HeLa, HEK-293 TPH-1	Activation of autophagy through inhibition of mTOR pathway by CPTP depletion;Enhancement of NLRP3 Inflammasome assembly in CPTP depletion state	Not determined in cell survival/death	[119]
17β-estradiol	Hepatocellular carcinoma (HepG2)	Activation of caspase-1-dependent pyroptosis;Inhibition of AMPK and activation of the mTOR pathway	Increased pyroptosis (amplified by autophagy inhibition)	[121]
	Thyroid cancer (Nthy-ori 3-1, BCPAP, BCPAP-ERα)	Activation of ROS-mediated autophagy in ERα-positive cell; Activation of the ERK1/2 pathway; promoting survival/growth of papillary thyroid cancer cells	Cancer cell survival	[122]

*P2X7* P2X purinoceptor 7, *ATG7* autophagy-related gene 7, *CPTP* ceramide-1-phosphate transfer protein, *ERα* estrogen receptor α

### Role of pathogen-mediated autophagy and inflammasome activation in cancer

Despite the links between infections and cancer, little is known about the molecular mechanisms underlying autophagy/inflammasome-mediated tumor progression in the context of an infection. As both autophagy and inflammasome pathways are strongly linked to innate immune system activation in response to various pathogens, numerous intracellular pathogens have developed strategies to escape from these responses [123]. Dysregulation of autophagy or inflammasomes may lead to defective host defense and harmful inflammatory responses during infection, exacerbating immunopathology [123]. In this session, we briefly discuss recent findings that the pathogen-mediated disturbance in the activation or inhibition of autophagy-inflammasome pathway and their consequences on the infection-associated initiation and progression of cancers.

HCV infection leads to the activation of inflammasomes and the production of pro-inflammatory cytokines, including IL-1α and IL-1β, thereby promoting inflammation, fibrosis, and carcinogenesis [124, 125]. HCV infection can cause progressive liver disease and hepatocellular carcinoma through NLRP3 inflammasome activation, which can be counteracted by autophagy activation under certain circumstances [124]. A recent study showed that HCV induced IRGM-mediated phosphorylation of ULK1 to facilitate viral genome replication, inducing autophagy [126]. The IRGM-mediated autophagy after HCV infection might contribute to the tumor-promoting effects of HCV.

*Helicobacter Pylori*, a pathogen linked to intestinal metaplasia and gastric cancer, can induce autophagy and inflammasome activation in the host [127–129]. Nevertheless, future studies are required to determine the relevance of autophagy/inflammasome activation in the carcinogenic effects of *H. pylori*. Additionally, human papillomaviruses (HPVs) suppressed IL-1β production in immortalized keratinocytes; the IL-1β production regulation was mediated by the HPV16 E6 oncoprotein at the post-translational level [130]. The inhibition of IL-1β production by HPV16 E6 may contribute to immune evasion and tumorigenesis [130]. Pathogen-mediated regulation of autophagy and inflammasome in connection with cancer are summarized in Table 3. A deeper understanding of the regulation of the inflammasome-autophagy axis by pathogens could enable the development of novel approaches to prevent or treat pathogen-associated cancers.

**Table 3:** Pathogen-associated regulation of autophagy and inflammasome in terms of carcinogenesis

Pathogen	Cell/tissue	Mechanism	Outcome	Ref.
Hepatitis C virus	THP-1, U2OS, Huh7, Huh7.5, K2040	NLRP3 inflammasome activation via calcium mobilization linked to phospholipase-C through HCV core protein	Increased inflammatory responses by HCV core protein	[125]
	Hepatocellular carcinoma (Huh7.25CD81)	Activation of autophagy through immunity-related GTPase M (IRGM)-mediated phosphorylation of ULK1	Promoting HCV replication by IRGM-mediated autophagy	[126]
*Helicobacter pylori*	Gastric cancer (AGS); murine primary gastric cell	Decrease of cathepsin-D sorting to the autophagosome in chronic exposure of VacA	Protective autophagy against *H. pylori*	[128]
	Bone marrow-derived macrophage, peripheral blood monocytic cell	NLRP3 inflammasome activation via potassium efflux, lysosomal destabilization, and increased ROS production by VacA and cagPAI of *H. pylori* virulence factor	Inflammasome-mediated adaptive immune response to control *H. pylori* infection	[129]
Human papillomavirus 16	Anogenital cancer, (CaSki, SiHa, HeLa, C-33 A), immortalized keratinocyte	Impaired IL-1β secretion by HPV16 E6 oncoprotein via post-translational control	Tumorigenesis	[130]

### Clinical trials assessing the anti-cancer effects of autophagy/inflammasome modulators

Several ongoing clinical studies are assessing the anti-cancer effects of modulators of either autophagy or inflammasome pathway. Much less is known about their potential usefulness in terms of the crosstalks between two processes. Nevertheless, recent and ongoing clinical trials of targeted approaches in the autophagy or inflammasome give us new insights into the exploration of dual regulation in designing anti-cancer therapeutics. Among the agents listed in Table 4, chloroquine and hydroxychloroquine are the most common autophagy inhibitors used as anti-cancer agents, mainly for the treatment of refractory cancers in combination with other chemotherapeutic agents or radiotherapy [131, 133–137]. Although some clinical trials were terminated early or showed negative results [138], numerous experimental and clinical studies showed promising effects in multiple myeloma, renal cell cancer, lymphoma, and pancreatic cancer regarding the use of autophagy inhibitors as anti-cancer agents (Table 4) [132, 135]. However, several questions remain unanswered regarding the selection of patients to examine the potential clinical usefulness of autophagy-targeting agents. The establishment of reliable and robust biomarkers, as well as dose optimization, would be imperative for the successful clinical development of autophagy modulators as anti-cancer therapies.

**Table 4:** Clinical trials to evaluate the safety and efficacy of targeting autophagy in cancer

Cancer type	Autophagy-targeting drug	Combination therapy	Phase (status)	Primary outcomes/results	Ref. or trial ID
Colon cancer	Hydroxychloroquine	FOLFOX/Bevacizumab	II (completed)	Response rate	NCT 01206530
	Hydroxychloroquine	Capecitabine/Oxaliplatin/Bevacizumab	II (completed)	Progression-free survival	NCT 01006369
Lung cancer	Chloroquine	none	I (terminated)	Incidence of adverse events	NCT 00969306
	Hydroxychloroquine	Paclitaxel/Carboplatin/Bevacizumab	II (completed)	Response rate	NCT 01649947
Breast cancer	Hydroxychloroquine	Letrozole/Palbociclib	I/II (recruiting)	Change in tumor proliferation index (Ki-67)	NCT 03774472
	Chloroquine	None	II (completed)	Response rate	NCT 01023477
	Chloroquine	None	II (completed)	Tumor proliferation index (Ki-67) : no difference	[131]
Multiple Myeloma	Hydroxychloroquine	Bortezomib	I (completed)	Very good PR: 14% Minor response: 14% SD: 45%	[137]
	Ricolinostat	Bortezomib/Dexamethasone	I/II (completed)	ORR: 37%	[132]
Prostatic cancer	Pantoprazole	Doxorubicin	I (completed)	PR: 8.3%	[133]
Renal cell cancer	Hydroxychloroquine	Aldesleukin	I/II(completed)	CR 10.3%, PR 10.3% SD 48.3%, PD 31.0%	NCT 01550367
Lymphoma	Hydroxychloroquine	Doxorubicin	I (in dogs) (completed)	ORR: 93.3% PFS: 5 months	[134]
Pancreatic cancer	Hydroxychloroquine	Gemcitabine/Paclitaxel	II (completed)	ORR: 38.2% (control 21.1%)	[135]
	Hydroxychloroquine	Gemcitabine	I/II (completed)	Decrease in CA 19-9: 61%	[136]
Glioblastoma	Hydroxychloroquine	Radiation/Temozomide	I/II (completed)	Overall survival	NCT 00486603

*FOLFOX* folinic acid (leucovorin), fluorouracil (5-FU), and oxaliplatin (Eloxatin); *ORR* overall response rate; *CR* complete remission; *PR* partial response; *SD* stable disease; *PD* progressive disease; *PFS* progression-free survival; Trial ID registered number at ClinicalTrials.gov

Inflammasome/pyroptosis-modulating agents have also been investigated for their anti-cancer and anti-metastatic effects in a clinical setting. CANTOS (Canakinumab Anti-inflammatory Thrombosis Outcomes Study) showed that canakinumab, a human monoclonal antibody targeting IL-1β, reduced the incidence of lung cancer in patients with atherosclerosis, highlighting it as a promising approach to prevent lung cancer [139]. Based on CANTOS study, phase 2 and 3 studies are underway to validate the efficacy of canakinumab as an adjuvant or neo-adjuvant treatment in lung cancer (Table 5). The anti-cancer effects of the inflammasome inhibitors anakinra and thalidomide have been tested in numerous cancers including colon cancer, breast cancer, multiple myeloma, prostate cancer, and pancreatic cancer (Table 5) [142, 143, 145]. In lines with the ongoing clinical trials (Table 5), either inflammasome inhibitor monotherapy or in synergy with other anticancer therapy should be confirmed in future investigations. In addition, various drugs including P2X purinoceptor 7 (P2X7R)-antagonist, andrographolide, and dibenzylideneacetone have been tested as a therapeutic strategy targeting inflammasome in multiple cancers (summarized in Table 5) [140, 141, 144, 146–149]. Clinical studies showed that the combination of thalidomide with cytotoxic chemotherapy provided durable responses in patients with refractory multiple myeloma, while the combination of docetaxel and thalidomide prolonged the overall survival in metastatic androgen-independent prostate cancer patients [142, 145]. Thus, the combination of inflammasome modulators with conventional chemotherapeutic drugs could provide synergistic anti-tumor effects in patients with advanced cancer, including those with chemoresistant or metastatic disease. Future randomized phase III clinical trials are required to confirm the anti-tumor effects and safety of these inflammasome-targeting compounds.

**Table 5:** Clinical and preclinical trials for anti-cancer strategies based on the regulation of inflammasome

Cancer type	Agent/drug	Combination therapy	Phase (status)	Primary outcomes/results	Ref. or trial ID
Colon cancer	Anakinra (IL-1β)	LV5FU2/Bevacizumab	II (completed)	Response rate	NCT 02090101
	Canakinumab (IL-1β)	Immune checkpoint inhibitor	I (ongoing)	Incidence of adverse events	NCT 02900664
	P2X7R antagonist	None	N/A	P2X7R: prognostic indicator & therapeutic target	[140]
Lung cancer	Canakinumab (IL-1β)	None	III (completed)	Total cancer mortality: HR 0.49 [95% CI 0.31–0.75]; *p* = 0.0009, Lung cancer incidence: HR 0.33 [95% CI 0.18-0.59]; *p* < 0.0001	[139]
	Canakinumab (IL-1β)	None	III (recruiting)	Effect of adjuvant treatment: Disease-free survival	NCT 03447769
	Canakinumab (IL-1β)	Pembrolizumab	II (recruiting)	Effect of neo-adjuvant treatment: Major pathologic response	NCT 03968419
Breast cancer	Anakinra (IL-1β)	Paclitaxel, Capecitabine, Eribulin, Vinorelbine	I (unknown)	Incidence of adverse events	NCT 01802970
	Andrographolide (NF-κB)	None	N/A	Inhibition of bone metastasis	[141]
Multiple myeloma	Anakinra (IL-1β)	Dexamethasone	II (completed)	20% partial response 16% minor response PFS : 37.5 months	[142]
	Thalidomide (caspase-1)	None	II (completed)	1 year event free survival: 22 ± 5% 1 year overall survival rate: 58 ± 5%	[143]
	Andrographolide (NF-κB)	None	N/A	Inhibition of myeloma cell proliferation	[144]
Prostate cancer	Thalidomide (caspase-1)	Docetaxel	II (completed)	PFS : 5.9 months (Docetaxel alone 3.7 months)	[145]
Lymphoma	BOT-4-one (NLRP3)	None	N/A	Inhibition of cell survival by inducing apoptosis	[146, 147]
Pancreatic cancer	Thalidomide (caspase-1)	Docetaxel	I (completed)	Maximum tolerated dose	NCT 00049296
	Dibenzylideneacetone (NLRP3)	None	N/A	Inhibition of growth and metastasis via impairment of chemotaxis	[148, 149]

*LV5FU2* 5-flourouracil and leucovorin, *HR* hazard ratio, *CI* confidence interval, *PFS* progression-free survival, *DBA* dibenzylideneacetone, trial ID registered number at ClinicalTrials.gov

A recent preclinical study is disappointing in demonstration of the findings that tumor-intrinsic PD-L1-NLRP3 inflammasome activation promoted resistance to anti-PD-L1 immunotherapy in multiple tumor models; this effect was mediated by the recruitment of granulocytic myeloid-derived suppressor cells into the tumor microenvironment, suppressing anti-tumor immune responses [150]. Despite this, multiple ongoing trials are investigating the anti-tumor effects of the combination of anti-PD1 immune checkpoint blockade and canakinumab in colon cancer, breast cancer, and non-small cell lung carcinoma (summarized in Table 5). Thus, it should be noted that increased sensitivity to immunotherapy, and immune checkpoint blockade in particular, is associated with inflammasome activation-associated gene expression profiles in melanoma patients [151]. Recently, a TMEM176B inhibitor and a newly identified negative regulator of NLRP3 inflammasome have been proposed as strategies to improve the efficacy of immune checkpoint blockade [151]. Although the use of inflammasome-related biomarkers has been suggested for monitoring immunotherapy responses, their usefulness needs to be confirmed in large patient cohorts. Future studies are warranted to determine how best to implement the current treatment options in combination with agents targeting the autophagy-inflammasome axis, in order to continue pursuing novel anti-cancer therapeutic discovery.

## Conclusions

Host immune responses to pathogens trigger both autophagy induction and inflammasome activation as a part of an innate immune response, aimed at combating the invading infectious agents. Similar to pathogens, tumor cells hijack host defense mechanisms, including autophagy and inflammasome activation, in an effort to escape immune surveillance. Dysregulation of autophagy or inflammasome activation is frequently observed in human cancers, potentially contributing to tumor progression. Nevertheless, the activation of these pathways could also suppress tumor growth, depending on the cancer type, disease stage, and tumor microenvironment. It is widely accepted that autophagy prevents excessive activation of inflammasomes; however, complex interactions occur between autophagy and inflammasome pathways. Emerging evidence suggests that in cancer cells, both autophagy and inflammasome activation are regulated by multiple cellular and biochemical cues, including mitochondrial ROS, autophagy/mitophagy-related molecules, PKR, and TRIM family members. Additionally, the communication between autophagy and inflammasomes may occur at specific intracellular organelles, such as MAMs, where signaling pathways define whether autophagy/inflammasome activation promotes or suppresses tumor growth.

It has become evident that modulation of the autophagy/inflammasome axis could provide clinical benefit in certain cancers, which are resilient to conventional treatments. Given the recent findings on the close relationship between autophagy and the inflammasome in several cancers, targeting both pathways might be a promising anti-cancer strategy. With the identification of an increasing number of autophagy/inflammasome modulators, we are just beginning to determine the potential clinical usefulness of autophagy/inflammasome-targeting to prevent or treat cancer. The clinical investigation of novel therapeutic interventions can be challenging, especially since the inflammasome and autophagy can have dual roles in cancer depending on the context. Besides, the crosstalk outcomes between the two pathways may be dynamic, depending on the tumor characteristics and the components of the tumor microenvironment. Thus, the development of personalized autophagy/inflammasome-targeting approaches might be required.

## Data Availability

Not applicable

## Abbreviation

AIM2: Absent in melanoma 2
AMPK: 5′-AMP-activated protein kinase
ASC: Apoptosis-associated speck-like protein containing a CARD
BCL2L13: BCL2-like 13
BNIP3: BCL2/adenovirus E1B 19 kDa protein-interacting protein 3
BNIP3L: BCL2/adenovirus E1B 19 kDa protein-interacting protein 3-like
dsDNA: Double-stranded DNA
ER: Estrogen receptor
FKBP8: FK506-binding protein 8
FUNDC1: FUN14 domain-containing 1
GSDMD: Gasdermin D
HMGB1: High-mobility group box 1
IL: Interleukin
IP3R: Inositol 1,4,5-trisphosphate (IP3) receptors
IRGs: Immunity-related GTPases
MAM: Mitochondria-associated membranes
mTOR: Mammalian target of rapamycin
NBR1: Neighbor of BRCA1
NDP52: Nuclear dot protein 52
NF-κB: Nuclear factor kappa-light-chain-enhancer of activated B cells
NLR: NOD-like receptor
NLRP3: NOD-, LRR-, and pyrin domain-containing protein 3
NRF2: Nuclear factor erythroid 2 (NFE2)-related factor 2
OMM: Outer mitochondrial membrane
OPTN: Optineurin
PD-L1: Programmed death-ligand 1
PE: Phosphatidylethanolamine
PI3K: Phosphoinositide 3-kinase
PI3P: Phosphatidylinositol 3-phosphate
PINK1: PTEN-induced putative kinase 1
PKR: Protein kinase RNA-activated or double-stranded RNA (dsRNA)-dependent protein kinase
RAGE: Receptor for advanced glycation endproducts
ROS: Reactive oxygen species
SNARE: Soluble NSF attachment proteins (SNAP) receptors
TLR: Toll-like receptor
TNFR: Tumor necrosis factor receptor
TRIM: Tripartite motif-containing protein
ULK1: unc-51-like autophagy activating kinase 1
VDAC1: Voltage-dependent protein channel 1
